# Distinct Genetic Architectures for Male and Female Inflorescence Traits of Maize

**DOI:** 10.1371/journal.pgen.1002383

**Published:** 2011-11-17

**Authors:** Patrick J. Brown, Narasimham Upadyayula, Gregory S. Mahone, Feng Tian, Peter J. Bradbury, Sean Myles, James B. Holland, Sherry Flint-Garcia, Michael D. McMullen, Edward S. Buckler, Torbert R. Rocheford

**Affiliations:** 1 Institute for Genomic Diversity, Cornell University, Ithaca, New York, United States of America; 2 Department of Crop Sciences, University of Illinois, Urbana, Illinois, United States of America; 3 United States Department of Agriculture – Agricultural Research Service, Robert W. Holley Center for Agriculture and Health, Ithaca, New York, United States of America; 4 United States Department of Agriculture – Agricultural Research Service and Department of Crop Science, North Carolina State University, Raleigh, North Carolina, United States of America; 5 United States Department of Agriculture – Agricultural Research Service and Division of Plant Sciences, University of Missouri, Columbia, Missouri, United States of America; 6 Department of Plant Breeding and Genetics, Cornell University, Ithaca, New York, United States of America; 7 Department of Agronomy, Purdue University, West Lafayette, Indiana, United States of America; The Wellcome Trust Centre for Human Genetics, University of Oxford, United Kingdom

## Abstract

We compared the genetic architecture of thirteen maize morphological traits in a large population of recombinant inbred lines. Four traits from the male inflorescence (tassel) and three traits from the female inflorescence (ear) were measured and studied using linkage and genome-wide association analyses and compared to three flowering and three leaf traits previously studied in the same population. Inflorescence loci have larger effects than flowering and leaf loci, and ear effects are larger than tassel effects. Ear trait models also have lower predictive ability than tassel, flowering, or leaf trait models. Pleiotropic loci were identified that control elongation of ear and tassel, consistent with their common developmental origin. For these pleiotropic loci, the ear effects are larger than tassel effects even though the same causal polymorphisms are likely involved. This implies that the observed differences in genetic architecture are not due to distinct features of the underlying polymorphisms. Our results support the hypothesis that genetic architecture is a function of trait stability over evolutionary time, since the traits that changed most during the relatively recent domestication of maize have the largest effects.

## Author Summary

Genetic architecture is of broad interest in evolutionary biology, plant and animal breeding, and medicine, because it influences both the response to selection and the success of trait mapping. Results from the most rigorously studied genetic systems suggest a similar genetic architecture across all species and traits studied, with many loci of small effect. A few strongly selected traits in domesticated organisms show unusual genetic architecture, for reasons that are unclear. We compare maize inflorescence, flowering, and leaf traits and show that inflorescence traits have distinct genetic architectures characterized by larger effects. Female inflorescences (ears) have larger effects than male inflorescences (tassels) even though the two structures have similar developmental origins. Analysis of pleiotropic loci shows that these larger effects are not inherent features of the underlying polymorphisms. Rather, maize inflorescences appear to be exceptionally labile, with female inflorescences more labile than male inflorescences. These results support the canalization hypothesis, which predicts that rapidly changing traits will have larger effects. We suggest that maize inflorescence traits, and ear traits in particular, have larger effects than flowering or leaf traits as a result of strong directional selection during maize domestication.

## Introduction

The genetic architecture of a complex trait is defined by the number, effect size, frequency, and gene action of the quantitative trait loci (QTL) that affect it. A comparison of studies from flies, mice, and humans shows that genetic architecture is remarkably consistent among these species, with many loci of small additive effect [1]. Distributions of QTL effect sizes are strikingly similar among different classes of mouse traits including behavior, biochemistry, immunology, and metabolism [2]. Similar results have been obtained in maize for flowering time, leaf morphology, and disease resistance traits [3]–[5]. Despite many well-powered genome-wide association studies (GWAS) of height variation in humans, no single polymorphism explaining even 1% of the variance in adult height has been found [6]–[9].

Fisher [10] provides a simple theoretical justification for these observations. For a well-adapted organism close to its fitness optimum, only small effects can increase fitness. Orr [11] showed that regardless of the distance from the fitness optimum, the expected distribution of effect sizes progressively fixed during adaptation is exponential, with a small number of large-effect loci fixed first, followed by progressively larger numbers of loci with smaller effects becoming fixed. The genetic architecture of intraspecific variation consists of many loci with small effects because loci with larger effects tend to be only briefly polymorphic.

A few traits exposed to strong, recent selection show distinct genetic architectures not characterized by many loci of small additive effect. For inbred dogs, three loci explain 38% of the variance in body weight among diverse breeds [12], and a single nucleotide polymorphism (SNP) at the IGF2 locus in pigs explains 15–30% of the variance in muscle mass [13]. In a cross between chicken populations recurrently-selected for high and low body weight, an epistatic network of four major loci explains 45% of the difference between parents [14]. Independent populations of anadromous stickleback fish that became trapped in freshwater lakes subsequently lost their armor plating through mutational changes at a single major locus [15]. The Fisher-Orr model predicts segregation of such large effects between populations exposed to divergent selective pressures, but not within a population exposed to directional selection.

Mating system also appears to influence genetic architecture. Flowering time QTL effects are much larger in the inbreeding species *Arabidopsis thaliana* than in maize, an outcrosser [16]. Inbreeding might allow isolated populations to fix large-effect mutations in response to divergent selective pressures, as the dog, chicken, and fish examples suggest. However, mating system differences cannot account for differences in genetic architecture between traits within an organism.

Plant and animal domesticates provide opportunities to compare genetic architecture between selected and unselected traits in populations exposed to the same demographic effects [17], [18]. Maize (*Zea mays* ssp. *mays*) was domesticated from teosinte (*Zea mays* ssp. *parviglumis*) 5,000 to 10,000 years ago in southwest Mexico [19]. Beadle [20] suggested that 4–5 recessive mutations underlie maize domestication as one out of every five hundred F_2_ progeny from a maize-teosinte cross appear maize-like. Two of these mutations have been identified: *teosinte branched1 (tb1)* causes an increase in apical dominance and reduction of lateral branching and *teosinte glume architecture (tga1)* causes “release” of the nutritious grain from bony, enclosing glumes [21], [22]. Other remarkable changes that occurred during maize domestication have yet to be fully explained.

**Figure 1:**
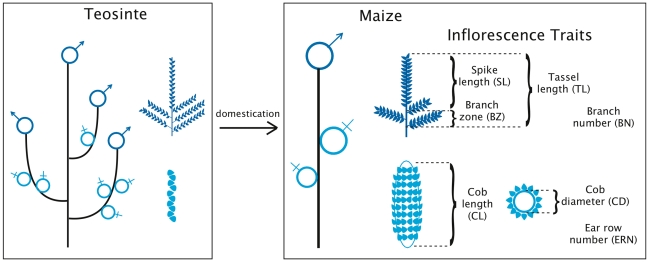
Evolution of plant and inflorescence architecture during maize domestication. Male inflorescences are depicted in dark blue, and female inflorescences in light blue. The seven inflorescence traits measured in this study are also depicted. Note the change in placement of the female inflorescence during maize domestication: it occupies multiple lateral positions on the primary lateral branch of teosinte, and occupies a single, apical position on the primary lateral branch of maize.

Maize is a monoecious plant with an apical male inflorescence, the tassel, and an axillary female inflorescence, the ear (Figure 1). Maize and teosinte tassels are relatively similar, but dissimilarity between maize and teosinte ears fueled historical controversy about whether one could have evolved from the other [23], [24] until molecular data provided irrefutable evidence that maize evolved from teosinte [19]. Teosinte “ears” are small, occupy the lateral positions of a primary lateral branch, and have two rows of kernels. Maize ears are large, occupy the apical position of a primary lateral branch, and have from eight to over twenty rows of kernels. Although maize tassels are clearly different from teosinte tassels, the maize ear stands out as a monument of morphological evolution under human selection.

The maize tassel and ear, despite their differences, share a common developmental origin and are nearly indistinguishable from each other during early development. Tassel and ear become distinct through the formation of long branch primordia and the abortion of female floral organs in the tassel, and through the abortion of male floral organs in the ear. Several mutant phenotypes support a close developmental relationship between tassel and ear. Branches are usually only found in the tassel, but a number of mutations produce branched ear phenotypes [25]. *Tasselseed* phenotypes are characterized by failure to abort female development in the tassel, and can be induced by mutation or epigenetic change [25], [26]. Because the underlying genetic control of maize tassel and ear development is so similar, human selection for ear morphology may have indirectly changed the morphology of the tassel as well.

In this study we compare the genetic architecture of thirteen maize morphological traits, including seven inflorescence traits reported here and three leaf and three flowering traits reported previously [3], [4]. The four tassel and three ear traits were measured over eight environments in the maize nested association mapping (NAM) population, a set of 4892 recombinant inbred lines (RILs) derived from 26 biparental families that capture much of the genetic diversity of maize [27]. These RILs are ∼97% homozygous, show little evidence for segregation distortion or inter-chromosomal linkage disequilibrium (LD), and have been genotyped with 836 markers for an average of one marker every ∼1.3 cM [28]. Two methods were used to detect QTL: linkage mapping across the 26 families (joint linkage) and a GWAS approach that incorporates polymorphism data from 1.6 million maize SNPs [4], [29]. The NAM population has been recently studied for flowering, leaf, and disease-resistance traits [3]–[5], revealing genetic architectures characterized by many loci of small additive effect. Maize inflorescence traits have distinct genetic architectures characterized by larger QTL effect sizes. Increased effect size in maize inflorescences is caused by many hundreds of polymorphisms with larger effects and a deficiency of small-effect inflorescence polymorphisms. Ear traits have the largest effects and also show lower model predictive abilities. The close developmental relationship between male and female maize inflorescences allows us to infer from our results that genetic architecture may vary independently of genetic control, providing new evidence for how selection affects the genetic architecture of complex traits.

## Results

### Maize inflorescence variation in a large, diverse population of recombinant inbred lines

The four tassel traits and three ear traits measured are shown in Figure 1 and in Table 1. Traits measured in units of counts, branch number (BN) and ear row number (ERN), were not normally distributed and required box-cox transformation. Traits measured in units of length, tassel length (TL), spike length (SL), length of the branch zone (BZ), cob length (CL), and cob diameter (CD), were normally distributed. Broad-sense heritabilities ranged from 0.87–0.93, within the range of heritabilities reported previously for flowering and leaf traits. Correlations between phenotypes from temperate and tropical growing environments were high for all inflorescence traits, so a single best linear unbiased predictor was calculated for each trait over all locations.

**Table 1:** Summary of maize inflorescence phenotypes and QTL results.

Class	Trait (abbreviation)	H^2^	Joint linkage QTL	GWAS SNPs (RMIP>4)
Tassel	Tassel length (TL)	0.93	37	241
Tassel	Spike length (SL)	0.92	33	286
Tassel	Branch zone (BZ)	0.92	26	303
Tassel	Branch number (BN)	0.94	39	325
Ear	Cob length (CL)	0.87	26	233
Ear	Cob diameter (CD)	0.90	39	317
Ear	Ear row number (ERN)	0.89	36	261

All traits were measured in 8 environments, and best linear unbiased predictors (BLUPs) were used to detect joint linkage QTL and GWAS SNPs.

### Genomic regions controlling maize inflorescence variation

We mapped loci controlling maize inflorescence variation using joint linkage and GWAS analyses in the NAM population of ∼5000 RILs as described previously [3]–[5]. Table 2 presents the major differences between these analyses, and full results are presented in Tables S1 and S2. In brief, the joint linkage analysis used 836 markers, whereas the GWAS analysis incorporated genetic information from over 1.6 million SNPs genotyped in the 27 parental lines. Joint linkage QTL were fit as marker-by-family terms, meaning that 26 separate effects were fit for each QTL [3], whereas GWAS SNPs are biallelic. A single joint linkage model was developed for the entire genome, whereas GWAS models were fit for each chromosome separately. For the GWAS analysis, a subsampling procedure was used to assign a resample model inclusion probability (RMIP) value for each SNP ranging from 0 to 1, representing the percentage of subsamples in which that SNP was selected [30]. High correlations were observed between trait heritabilities, the number of joint linkage QTL detected, and the number of GWAS SNPs detected across the seven inflorescence traits (Table 1). Full results for joint linkage and GWAS analyses are presented in Tables S1 and S2. GWAS analysis confirms the presence of all QTL detected by joint linkage analysis, and often splits a multiallelic QTL into two or more biallelic loci. The specific families assigned to carry a given QTL are often different between the two analyses.

**Table 2:** Comparison of the two methods used for genetic analysis.

	Joint Linkage (JL)	Genome-wide association (GWAS)
Model	Phenotype = mean+family+sum(QTL*family effects)+error	Phenotype = mean+family+sum(SNP effects)+error
Genetic information	836 SNPs scored in 4892 RILs	836 SNPs scored in 4892 RILs, and 1.6 million SNPs scored in 27 parental lines
Model fitting	Stepwise regression, whole genome simultaneously	Stepwise regression, each chromosome separately
Phenotypic data	Best linear unbiased predictors (BLUPs)	Residuals calculated from a joint linkage model excluding both the family effect and QTL on the chromosome under consideration
Significance threshold	alpha = .05, determined by 1000 permutations	alpha = .05, determined by 1000 permutations
Number of effects per QTL/SNP	26	1
Effect direction(s) of each QTL/SNP	May be both positive AND negative	Either positive OR negative
Resampling	None	100 subsamples, each composed of 80% of the RILs in each family sampled without replacement

Joint linkage mapping fits multi-allelic QTL across the entire genome, whereas the genome-wide association mapping method fits bi-allelic SNPs to one chromosome at a time.

### Inflorescence traits have larger QTL effects than flowering and leaf traits

**Figure 2:**
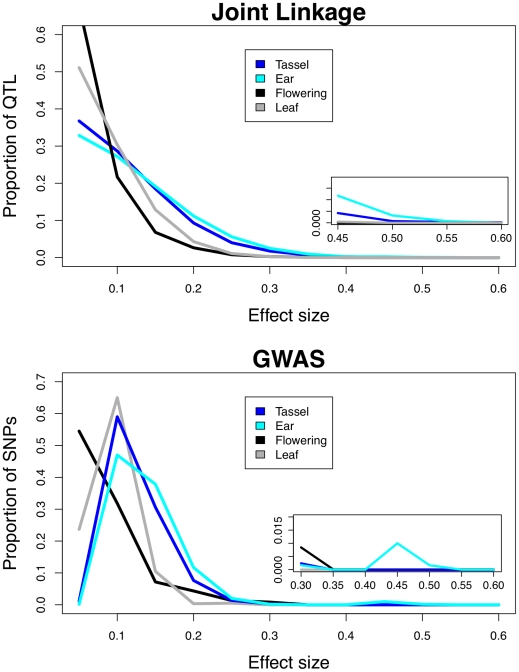
Frequency distributions of QTL effects. Effects from joint linkage (top) and GWAS (bottom) analyses are grouped by trait category (tassel, ear, flowering, and leaf). Effects for each phenotype are scaled by the total heritable variance (V_p_ * H_2_) in a panel of 282 diverse maize lines. Insets show the largest effects. Tassel traits include tassel length, spike length, branch zone, and branch number; ear traits include cob length, cob diameter, and ear row number; flowering traits include days to anthesis, days to silking, and anthesis-silking interval; leaf traits include leaf length, leaf width, and leaf angle.

We compared effects between ear, tassel, leaf, and flowering traits, and found that ear effects are largest and flowering effects are smallest in both joint linkage and GWAS analyses (Figure 2). Joint linkage analysis produces many more small effects than GWAS analysis as an artifact of the model fitting process, which assigns a separate effect to all 26 families at each QTL. Since most QTL are not present in all families, many of these effects are near zero. To compare effects among traits, the absolute value of each effect was scaled by the total heritable variation for that trait. Total heritable variation was calculated as the standard deviation of the trait BLUPs among a set of 282 diverse maize lines (this set includes the 27 parental lines) multiplied by our broad-sense heritability estimate for that trait (Table 1). Using the standard deviation of the trait BLUPs among just the 27 parental lines gave very similar results. Trait heritabilities were not included in an initial scaling process, leading to a modest correlation between heritability and median effect size (r^2^ = 0.127 for joint linkage and r^2^ = 0.233 for GWAS). Scaling by the total heritable variation reduced this correlation considerably (r^2^ = 0.045 for joint linkage and r^2^ = 0.075 for GWAS, Figures S1 and S2). QTL number varied from 26 to 40 among inflorescence, flowering, and leaf traits for joint linkage analysis. To control for variation in QTL number, we refit a model containing the 26 most significant QTL for all 13 traits and recalculated the effects. Results presented are for recalculated effects. This process did not change the magnitude of differences in effects among trait categories.

Our QTL effects are biased by the reference design of the NAM population, in which 26 diverse inbreds were each crossed to a common parent. Since the common parent is the reference point from which all other effects are judged, traits for which the common parent is an outlier, such as ear row number, will have inflated QTL effects in the NAM population. To correct for this bias, we inferred and present results of the full 26×26 matrix of QTL effects between all parental lines rather than using the 26×1 vector of observed QTL effects relative to the common parent (see Materials and Methods). We also regressed median QTL effects on the deviations of the common parent from the mean of the 27 parental lines and found little correlation (r^2^ = 0.067 for joint linkage and r^2^ = 0.043 for GWAS; Figures S1 and S2), even though ear row number had the largest deviation and the largest effects. To compare GWAS SNP effect sizes among traits, we calculated the absolute value of each median SNP effect across all subsamples in which that SNP was selected, and scaled this value by the total heritable variation. GWAS SNP number is correlated with heritability (Table 1), so we selected a fixed number of SNPs for each trait, ordered by decreasing RMIP value. Results presented at the bottom of Figure 2 include top 200 SNPs for each trait, and including the top 50, 100, or 500 SNPs yielded very similar results (Figure S3).

**Figure 3:**
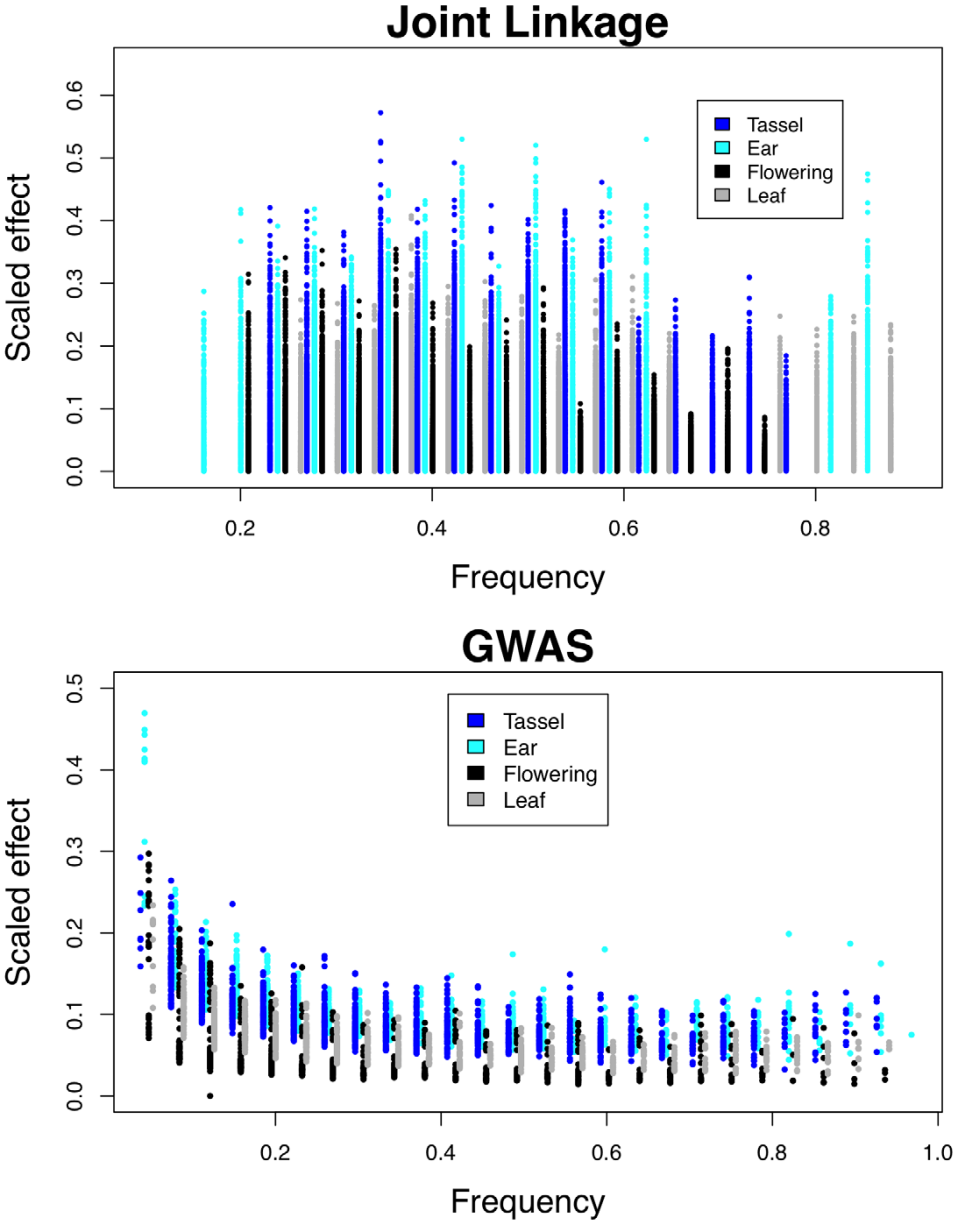
Relationship between QTL frequencies and effects. Effects from joint linkage (top) and GWAS (bottom) analyses are grouped by trait categories and scaled as in Figure 2.

Inflorescence traits have larger effects than flowering or leaf traits across a range of QTL and SNP frequencies (Figure 3; Kolmogorov-Smirnov test *p*<10^−16^ for joint linkage and GWAS), and ear traits have larger effects than tassel effects (Kolmogorov-Smirnov test *p* = 0.004 for joint linkage and *p*<10^−15^ for GWAS). The few large-effect flowering QTL are contributed by anthesis-silking interval (ASI; Figure S4), and not days to anthesis (DA) or days to silking (DS). Low-frequency GWAS SNPs present in four or fewer families account for nearly all loci with scaled effects above 0.15 (Figure 3). Several high-frequency, large-effect SNPs for ear row number are exceptions. The GWAS SNPs with the largest effects are found at low frequency and have low Resample Model Inclusion Probability (RMIP) values, although there is no overall correlation between frequency and RMIP (Figure S5). In contrast, joint linkage effect sizes show no relationship with frequency. Joint linkage results span a larger range of effect sizes than GWAS results, which likely reflects stacking of linked QTL with effects in the same direction.

### Model predictive ability is lower for ear traits

**Figure 4:**
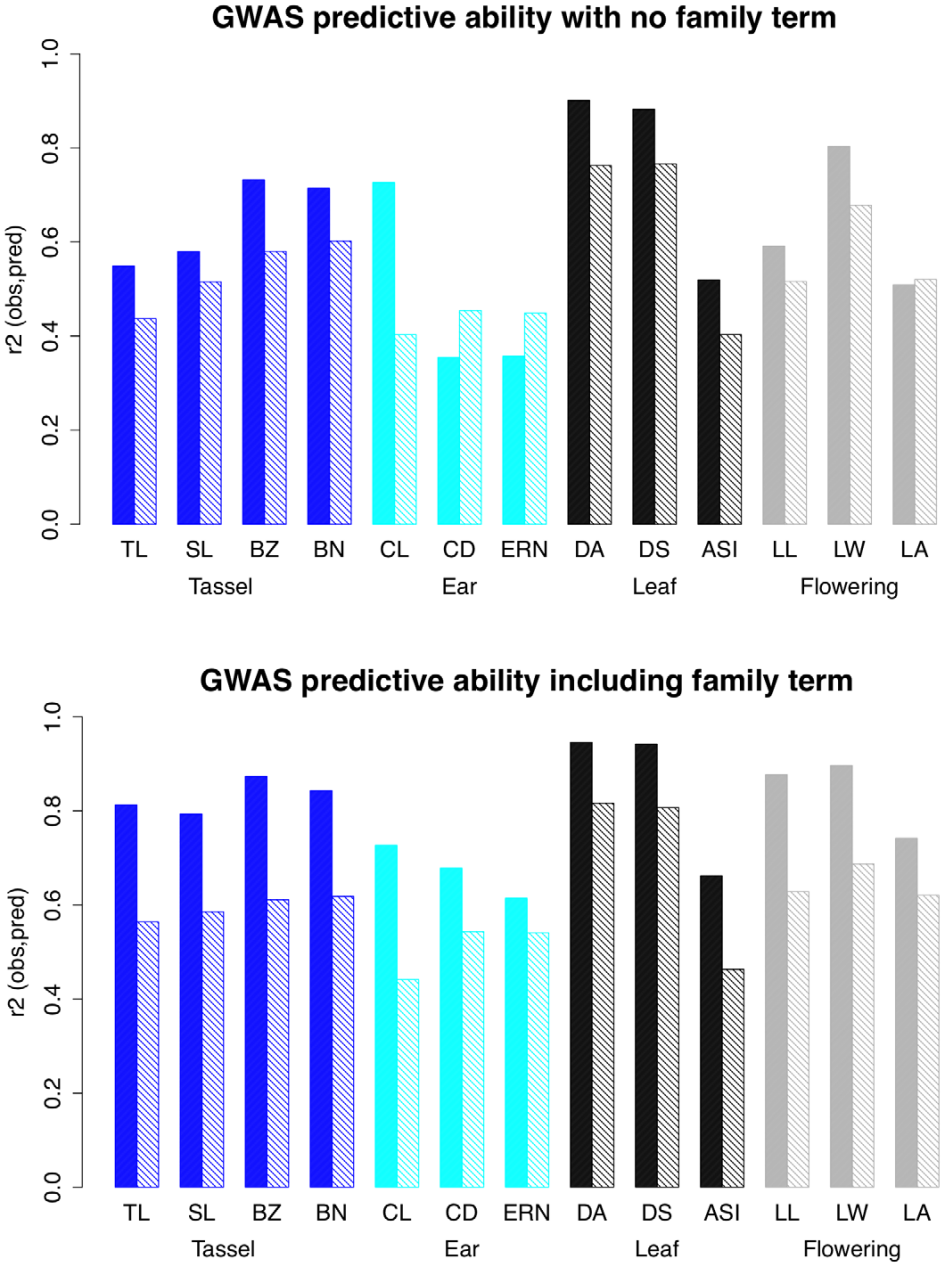
Predictive ability of GWAS models. The proportion of the phenotypic variance explained by GWAS models for 26 parental phenotypes (solid bars) and 4892 RIL phenotypes (hatched bars) in models without (top) and with (bottom) a family term. Trait categories are colored as in previous figures. TL = tassel length; SL = spike length; BZ = branch zone; BN = branch number; CL = cob length; CD = cob diameter; ERN = ear row number; DA = days to anthesis; DS = days to silking; ASI = anthesis-silking interval; LL = leaf length; LW = leaf width; LA = leaf angle.

We assessed the predictive value of our GWAS models for each trait by summing the effects of SNPs with RMIP values of 0.05 or greater, weighted by their RMIP values, and calculating predicted values for the 27 parents and the 4892 RILs, with and without the inclusion of a family term (Figure 4). Ear trait models had lower model predictive abilities than all other traits except anthesis-silking interval. Inclusion of the family term always improved predictive ability, and predictive ability was generally higher for parents than for RILs.

### Pleiotropic QTL affect ear and tassel traits

**Figure 5:**
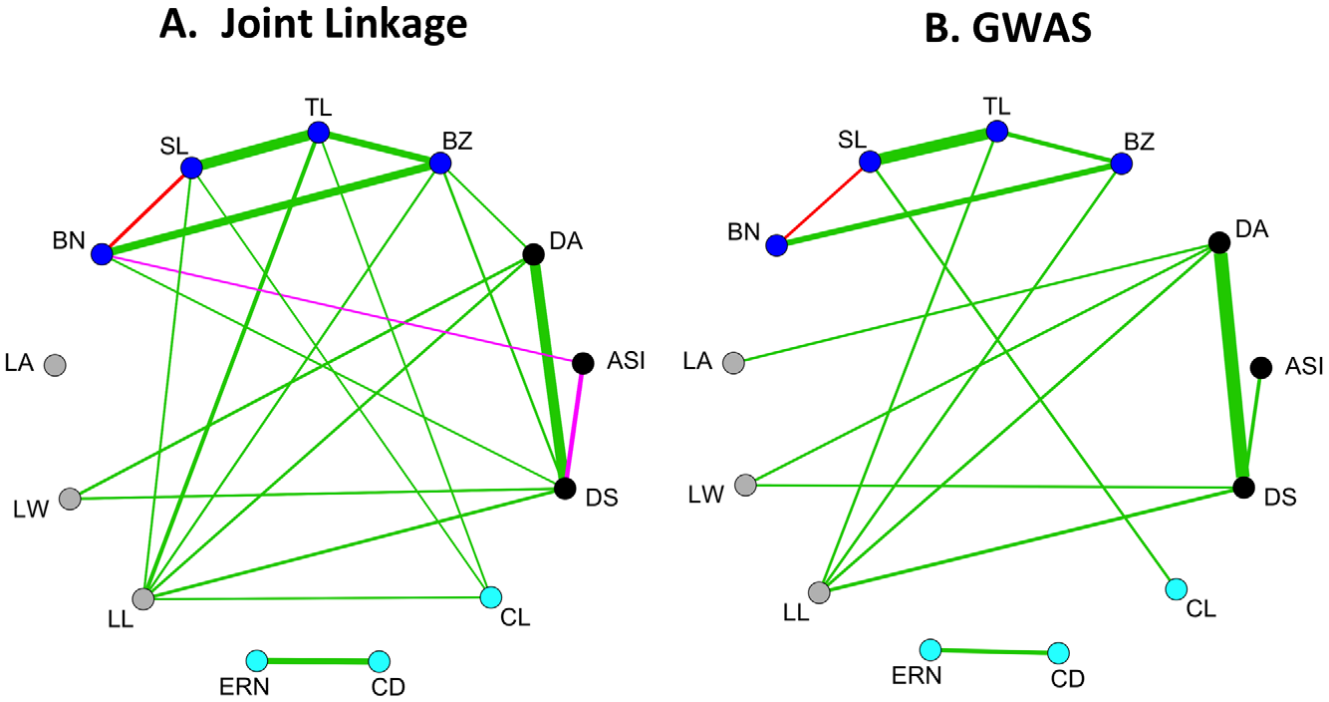
Pleiotropy for maize morphological traits. Green, red, and purple lines between traits indicate positive, negative, and both positive and negative correlations between QTL effects, respectively, with line width proportional to the degree of pleiotropy. A significance cutoff of *p*<0.01 (r>0.495 in a two-tailed test with 24 d.f.) was used for effect correlations. To eliminate spurious correlations, lines are only displayed for trait pairs with at least 10% pleiotropy. A. Pleiotropy assessed using joint linkage analysis. B. Pleiotropy assessed using GWAS. 5 cM sliding windows with a 2.5 cM step were used (see Materials and Methods), and only windows for which both traits had RMIP sums of at least 0.1 were considered. Trait abbreviations are the same as in Figure 4.

Joint linkage and GWAS analyses yield similar estimates of pleiotropy among 13 diverse maize morphological traits (Figure 5). Pleiotropy was assessed from joint linkage results by fitting the QTL for each trait to every other trait and correlating the resulting vectors of effects across the 26 families (Table S3, [3]). If a QTL has large positive or large negative effects for two traits in many of the same families, the effect vectors will be significantly correlated and pleiotropy will be inferred. For GWAS results, pleiotropy was assessed by averaging SNP effects for each trait in each family, weighted by their RMIP values, in sliding windows across the genome (see Materials and Methods and Table S4). Pleiotropy is observed between developmentally related traits across male and female inflorescences: cob length shows positive pleiotropy with spike length and with tassel length. Pleiotropy is also observed between elongation of vegetative and reproductive organs: leaf length shows positive pleiotropy with cob length, tassel length, spike length, and branch zone length. In addition we observed very strong pleiotropy between days to anthesis and days to silking, and moderate pleiotropy between both leaf length and leaf width with flowering traits. This pattern of pleiotropy has been observed previously using joint linkage results [3], [4] and is corroborated here using GWAS.

**Figure 6:**
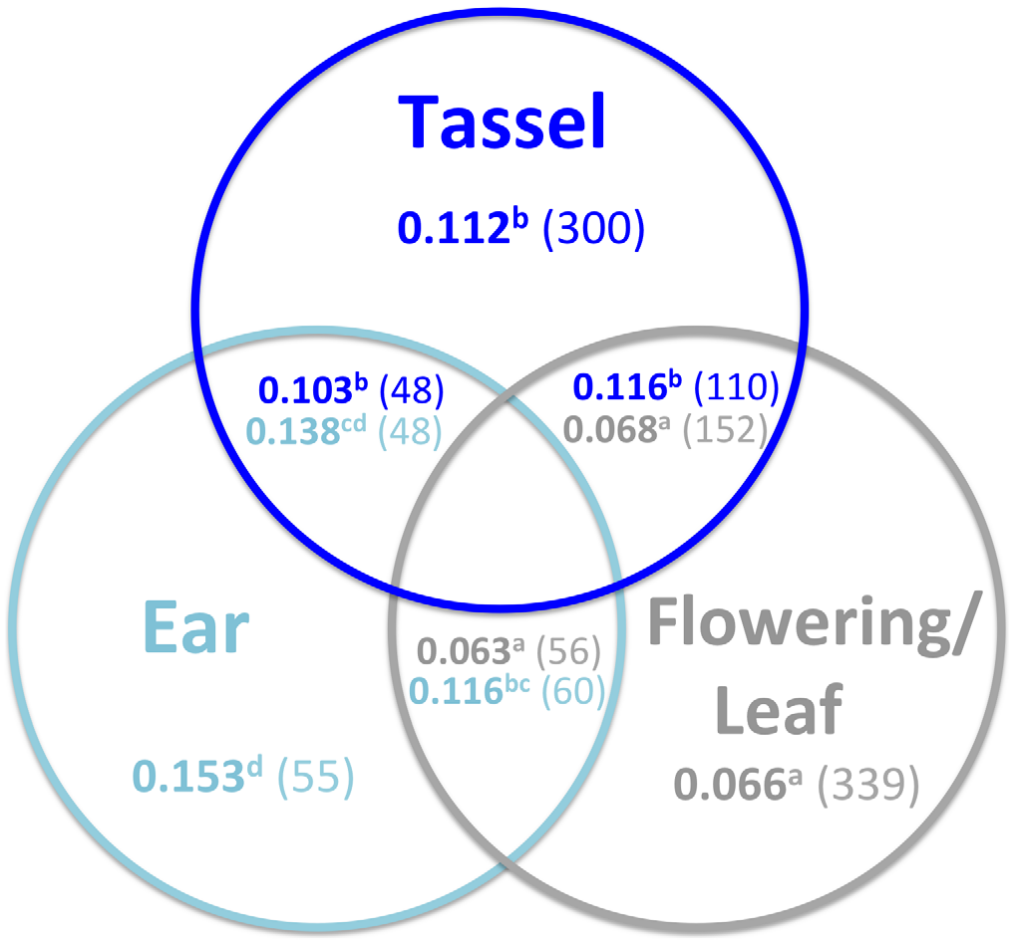
Relationship between QTL pleiotropy and effects. Mean effects (in bold) and SNP number (in parentheses) are shown for GWAS SNPs in sliding windows that show significant pleiotropy within and between trait categories. For simplicity, flowering and leaf traits are combined into a single category. Only pleiotropic SNPs are considered. Non-overlapping areas within circles represent instances of pleiotropy within a trait category (for example, pleiotropy between two tassel traits). In cases of pleiotropy between trait categories (overlap between colored circles), color is used to distinguish the mean effects and SNP numbers for the different trait categories. For example, in sliding windows in which significant pleiotropy is observed between tassel and ear traits, the 48 relevant tassel SNPs have a mean effect size of 0.103, whereas the 48 relevant ear SNPs have a mean effect size of 0.138. Superscripted letters indicate which effect distributions differ significantly from each other (Kolmogorov-Smirnov test *p*-value<0.05). Only the top 200 GWAS SNPs for each trait were included in the analysis, ordered by decreasing RMIP.

Since ear QTL have larger effects, we reasoned that the subset of QTL for other traits that show evidence of pleiotropy with ear traits might also have larger effects. To address this hypothesis, all pleiotropic GWAS SNPs were grouped according to whether they showed pleiotropy within or between trait categories (tassel, ear, and flowering/leaf; Figure 6). In general there are no differences in QTL effects between types of pleiotropic QTL within a trait category: pleiotropic tassel QTL have similarly-sized effects regardless of whether they are pleiotropic with ear, flowering/leaf, or other tassel QTL. The same pattern is observed for pleiotropic flowering and leaf QTL. The one exception is that ear QTL pleiotropic with flowering/leaf QTL appear slightly smaller than ear QTL pleiotropic with other ear QTL (Kolmogorov-Smirnov test *p* = 0.005).

When there is shared genetic control between ear traits and other traits, ear effects are larger than effects for other traits. Similarly, when there is shared genetic control between flowering/leaf traits and other traits, flowering/leaf effects are smaller than effects for other traits. Non-pleiotropic QTL are not displayed in Figure 6 but have significantly smaller effects than pleiotropic QTL, suggesting that our power to detect pleiotropy may be greater for QTL with larger effects.

### SBP–domain genes are enriched for proximity to tassel branching loci

Induced and spontaneous mutations in many maize genes cause dramatic inflorescence phenotypes (Table 3). We considered these genes to be candidates for our maize inflorescence QTL, and tested them for enriched proximity to our GWAS SNPs for maize inflorescence traits. Two of the genes responsible for changes in inflorescence morphology during maize domestication have also been identified: *teosinte glume architecture (tga1)* encodes a squamosa-binding-protein (SBP)-domain transcription factor [22] and *teosinte branched1 (tb1)* encodes a TCP-domain protein [21]. For this reason, annotated SBP-domain and TCP-domain genes in the maize genome were also considered to be candidates and tested for enriched proximity to our GWAS SNPs for maize inflorescence traits. To test for enrichment, we considered only the ten GWAS SNPs with the highest RMIP values for each trait, both to minimize the number of tests and because we assumed that these high-RMIP SNPs would be closely linked to their causal polymorphisms. For each of the three sets of candidates (26 genes identified using induced or spontaneous mutations, 17 SBP genes, and 24 TCP genes), we calculated the genetic distance to the nearest GWAS SNP for each gene and compared these results to a null distribution estimated from 1000 sets of the same number of random genes. For instance, the null distribution for SBP genes was estimated from 1000 sets of 17 random genes. Cloned maize inflorescence mutants showed slight enrichment for proximity to tassel length and spike length loci: GWAS SNPs for both these traits fell within 1 cM of the *fea2* and *td1* loci (Figure S6-top). SBP-domain genes showed enrichment for proximity to GWAS SNPs for branch number and branch zone length (Figure S6-middle). Overall, three SBP domain genes are implicated in tassel branching, at 4 Mb on chromosome 2, 205 Mb on chromosome 4, and 139 Mb on chromosome 10 (AGP version1 coordinates). The first of these genes corresponds to *liguleless1*, which lies near a high-RMIP SNP for leaf angle as reported by Tian *et al.* [4]. SBP genes have no overall enrichment for proximity to GWAS SNPs for leaf angle, however. TCP-domain genes show no significant enrichment for proximity to GWAS SNPs for any trait (Figure S6-bottom). Only the enrichment between SBP-domain genes and branch number survives a Bonferonni correction. Two well-characterized SBP-domain genes were included in our candidate list (*tga1* and *tsh4*), but are not associated with variation in branch number.

**Table 3:** Cloned maize mutants with inflorescence phenotypes and their co-localization with GWAS SNPs.

Gene	Homology (function)	chr	cM	Mb^1^	TL^2^	SL^2^	BZ^2^	BN^2^	CL^2^	CD^2^	ERN^2^	Ref
tasselseed2 (ts2)	alcohol dehydrogenase	1	62.7	146.81				49				[42]
barren inflorescence2 (bif2)	Kinase (auxin transport)	1	94.3	173.72								[43]
anther ear1 (an1)	Kaurene synth. (GA^3^ synth.)	1	141.9	240.50		34				100		[44]
teosinte branched1 (tb1)	TCP-domain TF^4^	1	159.4	264.85	7	12	14				5	[21]
Dwarf8 (D8)	DELLA (GA signaling)	1	159.7	265.20	7	12					5	[45]
knotted1 (kn1)	Homeobox	1	163.1	270.43					20		33	[46]
Indetermin. spikelet1 (ids1)	AP2-domain TF	1	191.1	291.83							10	[47]
*zea* floricaula/leafy1 (zfl1)	LEAFY	2	36.2	12.542					13			[48]
tasselseed1 (ts1)	Lipoxygenase (JA^5^ synth.)	2	69.9	45.65			7				14	[49]
Corngrass1 (Cg1)	miR156	3	29.1	7.60								[50]
ramosa2 (ra2)	LOB-domain TF	3	39.3	12.64	44							[31]
tasselseed4 (ts4)	miR172	3	63.9	141.07			72	42				[51]
terminal ear1 (te1)	RNA-binding	3	75.3	163.62		6	34				66	[52]
liguleless2 (lg2)	bZIP TF	3	85.3	175.05				36				[53]
barren stalk1 (ba1)	bHLH TF (auxin response)	3	93.3	181.36		15						[54]
sparse inflorescence1 (spi1)	YUCCA (auxin synthesis)	3	124.3	212.87				71	6	38		[55]
teosinte glume arch. (tga1)	SBP-domain TF	4	54.6	44.33					10	66		[22]
fasciated ear2 (fea2)	LRR receptor-like protein	4	58.8	132.74	79	88	67		74	7	58	[56]
thick tassel dwarf1 (td1)	LRR receptor-like kinase	5	63.5	60.96	85	105			5	80	48	[57]
Tassel sheath1 (Tsh1)	GATA zinc finger TF	6	104.9	166.17			9					[58]
ramosa1 (ra1)	C_2_H_2_ zinc finger TF	7	53.0	104.90	85			44	5			[59]
tasselsheath4 (tsh4)	SBP-domain TF	7	68.6	127.44	8							[60]
ramosa3 (ra3)	Trehalose 6-P phosphatase	7	109.6	161.17		103	71	50				[61]
branched silkless1 (bd1)	AP2-domain TF	7	123.1	166.49			10					[62]
delayed flowering1 (dlf1)	bZIP TF	7	134.8	169.75						9		[63]
zea floricaula/leafy2 (zfl2)	LEAFY	10	71.7	140.49				55		14		[48]

Genetic and physical positions are shown for 26 maize candidate genes with phenotypic effects on the inflorescence. Columns for each trait show the amount of GWAS signal within 1 cM of the gene.

1 midpoint of transcript (AGP version 1 coordinates).

2 Sum of RMIP (Resample model inclusion probability) values within 1 cM of candidate. Cells >100 indicate that some models contain <1 SNP per 2 cM window. TL = tassel length, SL = spike length, BZ = branch zone, BN = branch number, CL = cob length, CD = cob diameter, ERN = ear row number.

3 gibberellic acid.

4 transcription factor.

5 jasmonic acid.

## Discussion

### Low-frequency SNPs with very large effects may represent linked loci

Low-frequency SNPs found in four families or fewer account for most of the largest GWAS effects (Figure 3). Lack of power likely accounts for both the failure to detect small effect GWAS SNPs at low frequency and the greater proportion of intermediate-frequency GWAS SNPs relative to the null distribution (see [4] Fig. 4). Lack of power does not help explain the over-representation of large-effect SNPs at low frequency, however. Causal variants at low and high frequencies are more likely matched by random SNPs. A causal variant present in one or 25 of the 26 families has just 26 possible incidence patterns, whereas a causal variant present in 13 families has over 10 million possible incidence patterns. Our dataset of 1.6 million SNPs is too small to tag all causal variants, and we are far less likely to tag intermediate-frequency than low- or high-frequency variants. We observe large-effect SNPs at low frequency but not at high frequency, however. One explanation is linkage: linked variants with effects in the same direction will more often be combined into a single “synthetic” effect if they are present at low frequency. Low-frequency SNPs with very large effects also have low RMIP values (Figure S5), which supports this explanation: rare recombinant individuals allow separation of linked synthetic loci, but are sampled only intermittently. Because all GWAS SNPS with effects over 0.3 standard deviations in this study are found in a single family, we hypothesize that they result from linked QTL. These large effects explain a small proportion of the total phenotypic variation because their frequencies are low.

### Increased lability of the maize inflorescence

Larger QTL effects may reflect either larger effects of individual causal variants or greater linkage disequilibrium between causal variants with effects in the same direction. The latter phenomenon is expected to be most prevalent for SNPs found in a single family. However, the difference in magnitude between inflorescence and flowering/leaf effects holds true across the entire range of SNP frequencies (Figure 3), suggesting that individual inflorescence variants have larger effects than individual flowering or leaf variants. Also noteworthy is the deficiency of small effects for inflorescence variants, which cannot feasibly be due to linkage. Since many inflorescence traits are pleiotropic with flowering and leaf traits, we assume that many of the same polymorphisms underlie these QTL for different traits. Even in instances of shared genetic control, however, inflorescence effects are larger than flowering/leaf effects, and ear effects are larger than tassel effects (Figure 6). This does not support the scenario that inflorescence polymorphisms are unique, consisting for example of more frame-shifts, premature stop codons, or nonsynonymous substitutions. Rather, these results suggest that the maize inflorescence, and the maize ear in particular, is more labile.

### Other traits with distinct effect sizes

Three flowering traits show a disjunct distribution of effect sizes, with days to anthesis (DA) and days to silking (DS) effects much smaller than anthesis-silking interval (ASI) effects (Figure S4). Stabilizing selection over millions of years may have purged *Zea* populations of large-effect variants for DA and DS due to the fitness cost of flowering too early or late relative to the rest of the population. In contrast, ASI may be a much “younger” trait specific to the apically-dominant architecture of the maize plant. Our scaling procedures may also have inflated effects for ASI. The development and maintenance of inbred lines by self-fertilization strongly selects for synchronous male and female flowering (ASI values close to zero), reducing the total heritable variation in ASI and increasing our scaled ASI QTL effects.

### Reduced predictive ability of additive models for ear traits

The utility of GWAS studies is contingent on their ability to predict phenotypes. In this study we show that simple additive models containing several hundred SNPs explain over 50% of the phenotypic variation in a set of 4892 RILs for most of the 13 maize morphological traits (Figure 4). SNP number in these models could probably be reduced considerably without sacrificing predictive ability by removing SNPs in high linkage disequilibrium with each other [5]. Additive model predictions are least accurate for the ear traits (cob length (CL), cob diameter (CD), ear row number (ERN)), and the flowering trait anthesis-silking interval (ASI). To investigate the nature of this apparent non-additivity, we focus on models without a family term (Figure 4-top) that rely solely on GWAS SNPs to explain phenotypic differences within and between families. Most traits show ∼10% greater predictive ability in the parents than in the RILs, but for cob length this difference is dramatic (∼30%). We observe the opposite situation for cob diameter and ear row number: predictive ability is higher in the RILs than in the parents. Here we interpret these observations in terms of interaction effects. For cob length, additive effects detected in the RILs accurately predict parental phenotypes, so we infer that interaction effects are equally likely to enhance or mask a given QTL (their mean effect is close to zero) and they must be common enough to account for a ∼20% drop in predictive ability in the RILs. For cob diameter and ear row number, additive effects detected in the RILs do not predict parental phenotypes, so we infer that parental phenotypes are caused by more complex interaction effects that are seldom recapitulated in the RILs and have little influence on additive effect sizes.

### Pleiotropic loci affect elongation of leaves and inflorescences

We observe several pleiotropic relationships consistent with previous developmental genetic work. Negative pleiotropy between spike length (SL) and branch number (BN) indicates a trade-off between the two, consistent with the finding that a given meristem in the maize inflorescence acquires the fate of either a long indeterminate branch or a short indeterminate spikelet pair [31]. Knowledge of shared developmental networks, not only between ears and tassels but also between the elongation of vegetative and reproductive structures, can help inform the choice of candidate genes. The QTL with pleiotropic effects on leaf length, tassel length, and cob length may involve genes that function in cell elongation throughout the plant, rather than inflorescence-specific developmental genes.

### Distinguishing linkage from pleiotropy

In a biparental family, close linkage of genes cannot be distinguished from pleiotropic effects of a single gene. Assessment of pleiotropy in NAM is made possible by testing correlations between vectors of QTL or SNP effects across the 26 families of RILs. This analysis will only detect pleiotropy when the same polymorphism or haplotype is consistently associated with phenotypic effects on different traits. Another less stringent definition of pleiotropy would allow a single gene to control variation in different traits through different polymorphisms. A possible example of this type of pleiotropy is the *liguleless1 (lg1)* locus, which is associated with variation in both leaf angle [4] and tassel branching (this study). *lg1* encodes an SBP-domain transcription factor. The association of *lg1* with leaf angle is supported by its mutant phenotype [32], and the association of SBP-domain transcription factors with branching is supported by our results and by studies in rice [33], [34]. Effect estimates for *lg1*-linked leaf angle and branch number QTL in NAM are not correlated, suggesting that different polymorphisms may be responsible for the effects of *lg1* on leaf and tassel traits. Since structural mutations in a gene are more likely to have effects wherever the gene is expressed, *lg1*-linked variants for leaf angle and branch number might be *cis*-regulatory variants operating independently of each other in specific tissues [35]. Pleiotropy of this type cannot be distinguished from linkage in our analyses.

### Loci controlling natural variation in maize inflorescence traits are distinct from those uncovered using mutagenesis

Only a small degree of overlap is observed between the location of cloned maize inflorescence development genes and SNPs significant for inflorescence traits (Table 3, Figure S6A). Overlap between SBP-domain genes and loci for tassel branch number shows that our analysis has the power to detect such overlap where it does exist (Figure S6B). Most cloned maize inflorescence genes involve loss-of-function alleles generated by transposon or chemical mutagenesis that have obvious phenotypes in mutant screens. Such screens generally cannot uncover mutations for which there is genetic redundancy. Purifying selection may be relaxed for genes with redundant functions, allowing them to accumulate more mutations that change gene function than non-redundant genes. If this is true, then mutagenesis studies may be somewhat biased against the discovery of loci controlling natural variation.

### Effects of selection on genetic architecture

The genetic architecture observed for maize inflorescence traits is novel. Very large effect sizes for a few major loci are commonly observed in plant and animal domesticates, including maize-teosinte segregants [36], divergently-selected dog breeds [12] and chicken populations [14]. Fish populations subjected to habitat change [15] demonstrate that these unusual genetic architectures may be caused by natural as well as human selective pressure. These observations are consistent with theoretical predictions of an exponential distribution of effect sizes underlying adaptation [11]. In each case, the number of large-effect QTL is very few, because large effects quickly move a trait close to its fitness optimum. In contrast, the genetic architecture of inflorescence traits within domesticated *Zea* is characterized by a shift in the entire distribution of effect sizes, with many more effects of intermediate size and many fewer small effects. Although unusual genetic architectures observed in domesticates are sometimes attributed to human preference for novelty, which may preserve unadaptive, large-effect mutations [12], it is difficult to explain how such a preference for novelty could account for the deficiency of small-effect inflorescence QTL.

Maize domestication released cryptic genetic variation for inflorescence traits [37]. For example, ear row number is invariant in teosinte but varies widely in maize, indicating that all genetic variants for ear row number in maize must be cryptic genetic variants in teosinte. Maize inflorescence QTL may have more large effects and fewer small effects because more of them are caused by newly-released cryptic variants. The distribution of effects for cryptic variation could differ from that of old, standing variation for two reasons. First, large effects become fixed or purged more rapidly than small effects [38]. Second, large-effects could become smaller through the gradual accumulation of buffering mutations [39]. This is the canalization hypothesis: organisms evolve robustness to genetic and environmental perturbation. Since the maize ear is a relatively recent creation, it has accumulated the least genetic buffering. These scenarios differ in their prediction of the distribution of effects of new mutations: either large-effect mutations keep arising transiently, or the canalized phenotype becomes resilient to large-effect mutations.

Maize ear and tassel traits have distinct genetic architectures even though they have shared genetic control: pleiotropic loci with effects on both tassel and ear show larger effects on the ear. This is the expected pattern if these pleiotropic loci had phenotypic effects in male but not female inflorescences in teosinte. Following maize domestication, they would act as newly-released cryptic variants in maize ears but not tassels. Maize domestication moved the ear from an axillary position to an apical position in the primary branch, which may have brought it under the control of an apical dominance network [40].

The process of domesticating maize from teosinte transformed plant architecture. The long lateral branch of teosinte with multiple, axillary, two-rowed female inflorescences was reshaped into a short, unbranched structure bearing a single, apical, multi-rowed ear. We present evidence that this process also transformed genetic architecture, creating a state of increased genetic lability in the maize ear that humans have cleverly exploited. Because only a few thousand generations have elapsed since the maize ear was created, ear traits still show a larger range of effect sizes than tassel, flowering, and leaf traits, for which maize and teosinte are phenotypically much more similar.

Future advancements in medicine and agriculture will benefit from an improved understanding of the forces that shape the genetic architecture of complex traits. The most rigorous study to date comparing the genetic architecture of traits within a species [2] examined 97 traits in mice and found little variation in effect size (see [1] Figure 2). These traits were predominantly fitness-related and may have stabilized over many millions of years. By comparing a suite of maize morphological traits that have experienced very different selective pressures over the last 5,000 years, we show that effect sizes are inversely proportional to trait stability and that genetic architecture may vary even when there are common underlying genes. We suggest that most large-effect maize ear QTL represent cryptic genetic variants released by the fixation of large-effect domestication mutations. The release of cryptic variation by directional selection might help explain the seemingly inexhaustible genetic variation in long-term selection experiments [41]. Because transgenesis can have large effects, it may also unveil cryptic variants, suggesting that interaction between natural and transgenic variation could impact phenotypes and selection schemes for a variety of domesticated and agricultural organisms.

## Materials and Methods

### Plant materials and phenotypic evaluations

The creation of the NAM population of RIL families has been described previously [3], [28]. Environments, field design, traits. Another publicly-available maize RIL family, the intermated B73-by-Mo17 (IBM) family, was also included in our analyses for a total of 4892 RILs from 26 biparental families with B73 as a common parent, and a total of 27 parents. All inflorescence traits were measured in eight environments, including Aurora, NY, Clayton, NC, Urbana, IL, Homestead, FL, and Ponce, PR in 2006, and Aurora, NY in 2007. Tassel traits were additionally measured in Columbia, MO in 2006 and Urbana, IL in 2007. Ear traits were additionally measured in Clayton, NC in 2007 and Aurora, NY in 2008. In each location, each family was represented by 220 rows: 200 rows of RILs and 10 rows of each parent. Data from some RILs was later discarded to bring the total RIL number to 4892 [28].

### Phenotypic data transformation and best linear unbiased predictor (BLUP) calculation

Trait transformations were performed using the boxcox function in R with lambda ranging from −10 to +10 in increments of 0.1, where lambda values of 0 and 1 are equivalent to log and linear transformations, respectively. Branch number and ear row number traits had maximum likelihood values of lambda of 0.3 and 0.4 respectively. Box-cox transformed values of these traits were used to calculate BLUPs. BLUPs were calculated in SAS using PROC MIXED and a model with location, set(location), family, family*location, and entry(family) as random effects.

### Genotypic data and joint linkage analysis

The genotypic dataset consisted of 836 markers, representing the subset of 1106 markers that could be placed unambiguously on the physical map, scored on 4892 RILs. Missing data, consisting primarily of markers that were non-informative in particular families, were imputed as previously described [4]. Joint linkage models were obtained in SAS using the stepwise selection procedure in PROC GLMSELECT. The family term was forced into the model, and each of the 836 possible marker-by-family terms was made available for inclusion. Significance levels for entry and exit of model terms were determined by permutation: phenotypic data were permuted against the genotypic data separately within each family, all 836 marker-by-family terms were tested, and the lowest resulting p-value was recorded for each permutation. 1000 permutations were performed, and alpha was set at .05.

### SNP imputation, projection, and GWAS analysis

Missing SNP data from the maize HapMap project [29] were imputed as previously described [4]. For non-recombinant RIL marker intervals, SNP values of 0 (common parent allele) and 1 (alternate allele) were assigned according to the parental genotype. For recombinant RIL marker intervals, SNP values between 0 and 1 were assigned based on the physical position of the SNP within the interval and assuming a linear relationship between physical and genetic distance. Projection was also tested assuming a linear relationship between “genespace” and genetic distance, but this had very little effect on the results. GWAS models were fit for each chromosome separately. The phenotypes for each chromosome consisted of residuals from a joint linkage model excluding both the family covariate and all QTL on the chromosome under consideration. GWAS genotypes were obtained by scoring 1.6 million SNPs in the 27 parental lines and then “projecting” these genotypes into the progeny RILs. We employed a subsampling procedure wherein 80% of the RILs from each family were sampled without replacement, and forward regression was used to fit SNPs in the presence of the family term using permutation-derived significance thresholds [4]. This process was repeated 100 times to obtain a resample model inclusion probability (RMIP) value for each SNP ranging from 0 to 1, which represents the percentage of samples in which that SNP was selected. Only SNPs with RMIP values greater than or equal to 0.05 were used for further analysis.

### QTL effect sizes

QTL effects for each trait were divided by the standard deviation of BLUP values across a set of 282 diverse maize lines that included the 27 parental lines, and multiplied by the broad-sense heritability estimate for that trait. Since a minimum of 26 QTL were detected for each trait, a 26-QTL model was refit for each trait and used to determine effect sizes. This experiment used a reference design (26 inbred lines were each crossed to a common parent), meaning that QTL effect sizes are potentially biased for traits for which the common parent is an outlier. To circumvent this problem, for each QTL we calculated the predicted effects of all pairwise matings between the 26 parents (eg: for two parents with effects of +1 and −1 relative to the common parent, the predicted QTL effect size in this family is 2), yielding a total of 325 (26 choose 2) effect sizes for each QTL, or a total of 6825 qtl effects per trait.

### Pleiotropy

Pleiotropy between pairs of traits in the joint linkage analysis was evaluated as described previously [3]. Briefly, the QTL model for each trait was applied to every other trait, and correlations between effect estimates were used to detect significant pleiotropic QTL. For each QTL in each pairwise trait comparison, the Pearson correlation coefficient *(r)* between the two effect vectors of length 26 is significant at *p*<0.01 if *r* exceeds 0.495 (two tailed *t* distribution, 24 d.f.). The percentage of shared QTL between two traits is the sum of two fractions: the fraction of significant correlations when the model for trait 1 is applied to trait 2, and vice versa. Pleiotropy between pairs of traits in GWAS analysis has not been reported previously. First, the effects of all GWAS SNPs for each trait in each family were weighted by their RMIP values and averaged in sliding windows across the genome, in order to derive a vector of effect estimates for each trait in each window. Results presented here used a 5 cM window size and a 2.5 cM step, but similar results were obtained for larger and smaller windows. Second, for each pair of traits, only windows where the sum of RMIP values for each trait fell above a threshold (RMIP = 0.10 for the results presented) were considered. Finally, significance of Pearson correlation coefficients between effect estimates was calculated as for joint linkage analysis.

### Co-localization of QTL and candidate genes

We considered only the top ten GWAS SNPs for each trait, ordered by decreasing RMIP value, on the assumption that these more robustly-selected SNPs should be more closely linked to the causal variants. To test for significant enrichment, the number of high-RMIP SNPs for a given trait that fell within 0.5, 1, and 2 cM of candidates was compared with a null distribution obtained by selecting an equivalent number of random genes (eg: 17 random genes for comparison to 17 SBP candidates), calculating their proximity to trait SNPs, and repeating this process 1000 times. Selection of random positions rather than random genes represents a far less stringent test, since genes are clustered in the maize genome.

## Supporting Information

Figure S1 Median scaled effects of joint linkage QTL for 13 traits regressed on broad-sense heritability (A) and on B73's phenotypic standard deviation from the mean in a panel of 282 diverse maize lines (B).
(TIF)

Figure S2 Median scaled effects of GWAS SNPs for 13 traits regressed on broad-sense heritability (A) and on B73's phenotypic standard deviation from the mean in a panel of 282 diverse maize lines (B).
(TIF)

Figure S3 Frequency distributions for scaled GWAS effects produced using the top 50 (A), 100 (B), and 500 (C) SNPs for each trait. Results are similar to those presented at the bottom of Figure 2.
(TIF)

Figure S4 Relationship between QTL frequency and scaled QTL effects in the joint linkage (A) and GWAS (B) analyses, as in Figure 3 but with ASI effects colored in red.
(TIF)

Figure S5 Relationship between RMIP and scaled effects (top), and between SNP frequency and RMIP (bottom).
(TIF)

Figure S6 Co-localization of GWAS SNPs and candidate genes. A: cloned inflorescence mutants (n = 26). B: SBP-domain genes (n = 17). C: TCP-domain genes (n = 24). The 10 GWAS SNPs with the highest RMIP values were tested for each of 13 maize morphological traits. The number of GWAS SNPs falling within 0.5, 1, and 2 cM of a candidate gene is indicated with black, grey, and white bars respectively. Significance at levels of p<0.05 (*) and 0.01(**) was obtained by selecting an equal number of random maize genes to the number of candidates, calculating their genetic distances to the top 10 GWAS SNPs, and repeating this procedure 1000 times. Significance levels differ greatly between traits due to differences in the genetic context of their GWAS SNPs. SNPs in regions of low recombination may fall within 1 cM of many more random genes than SNPs in regions of high recombination.
(TIF)

Table S1 Joint Linkage QTL.
(XLS)

Table S2 GWAS SNPs.
(XLS)

Table S3 Pleiotropy in joint linkage analysis.
(XLS)

Table S4 Pleiotropy in GWAS analysis.
(XLS)
