# Supplementary figures and images for: Distinct Genetic Architectures for Male and Female Inflorescence Traits of Maize
Source: PLoS Genet. 2011 Nov 17;7(11):e1002383. doi: 10.1371/journal.pgen.1002383 (PMC3219606; doi:10.1371/journal.pgen.1002383)

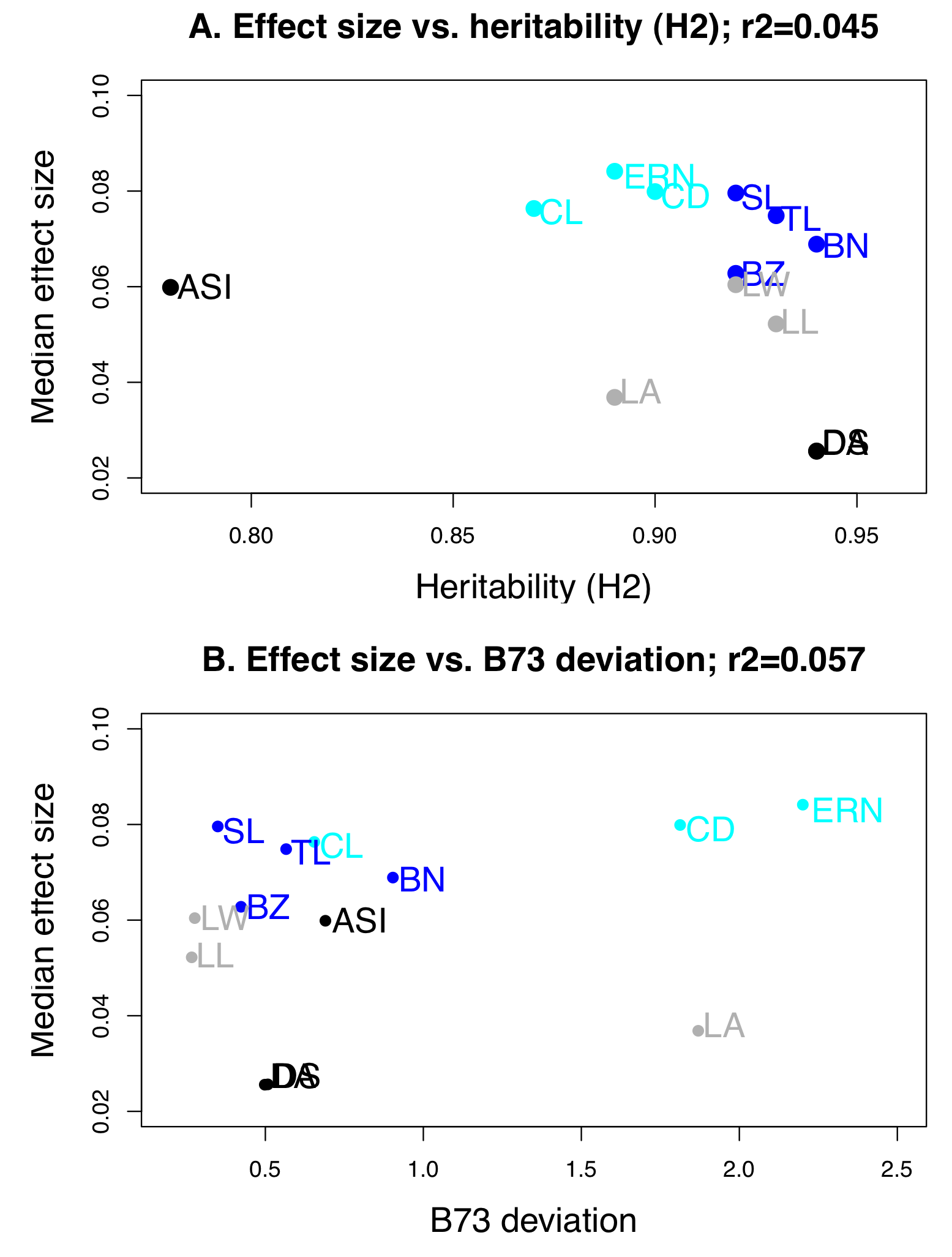

Supplement: Figure S1 — Median scaled effects of joint linkage QTL for 13 traits regressed on broad-sense heritability (A) and on B73's phenotypic standard deviation from the mean in a panel of 282 diverse maize lines (B). (TIF) [file pgen.1002383.s001.tif]

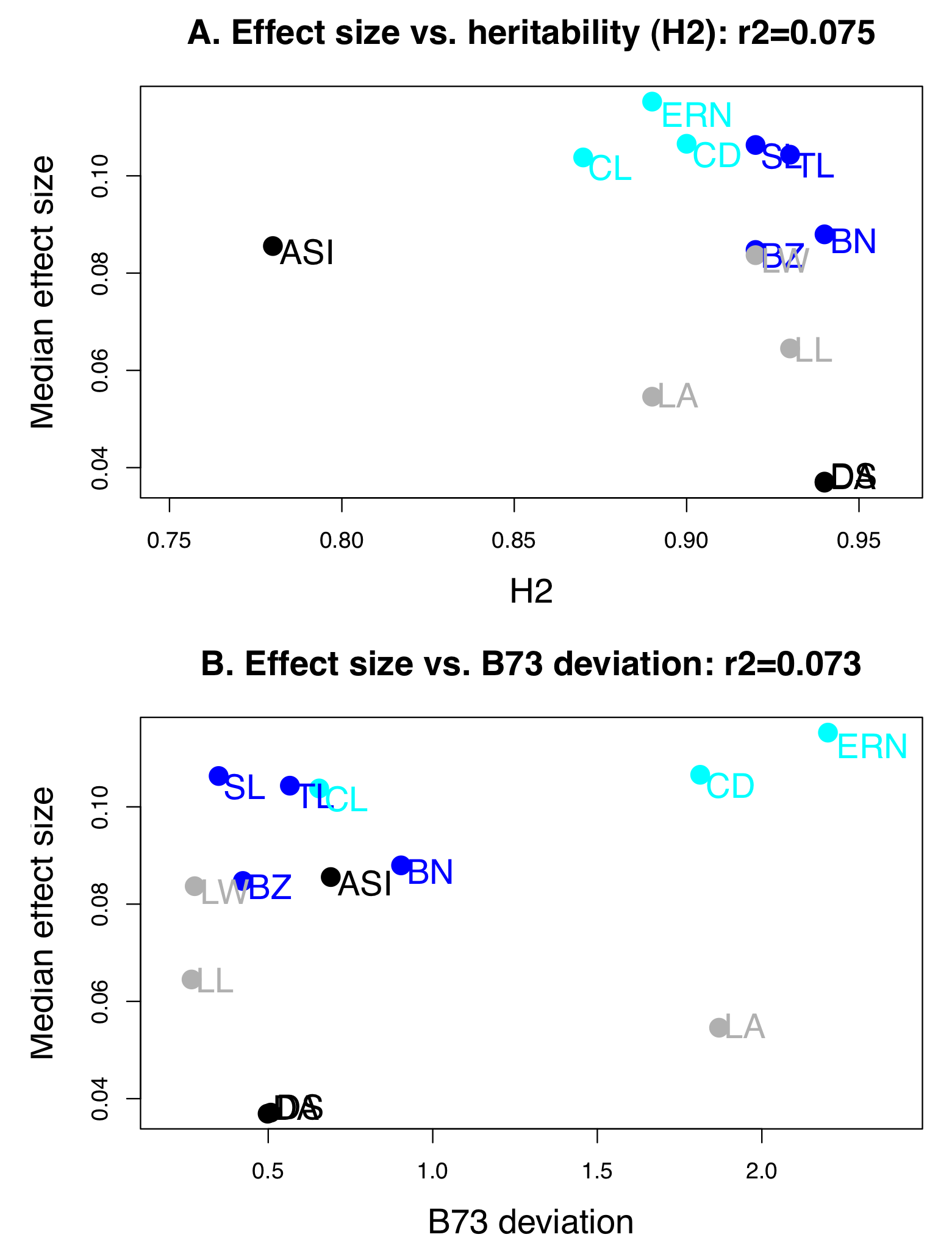

Supplement: Figure S2 — Median scaled effects of GWAS SNPs for 13 traits regressed on broad-sense heritability (A) and on B73's phenotypic standard deviation from the mean in a panel of 282 diverse maize lines (B). (TIF) [file pgen.1002383.s002.tif]

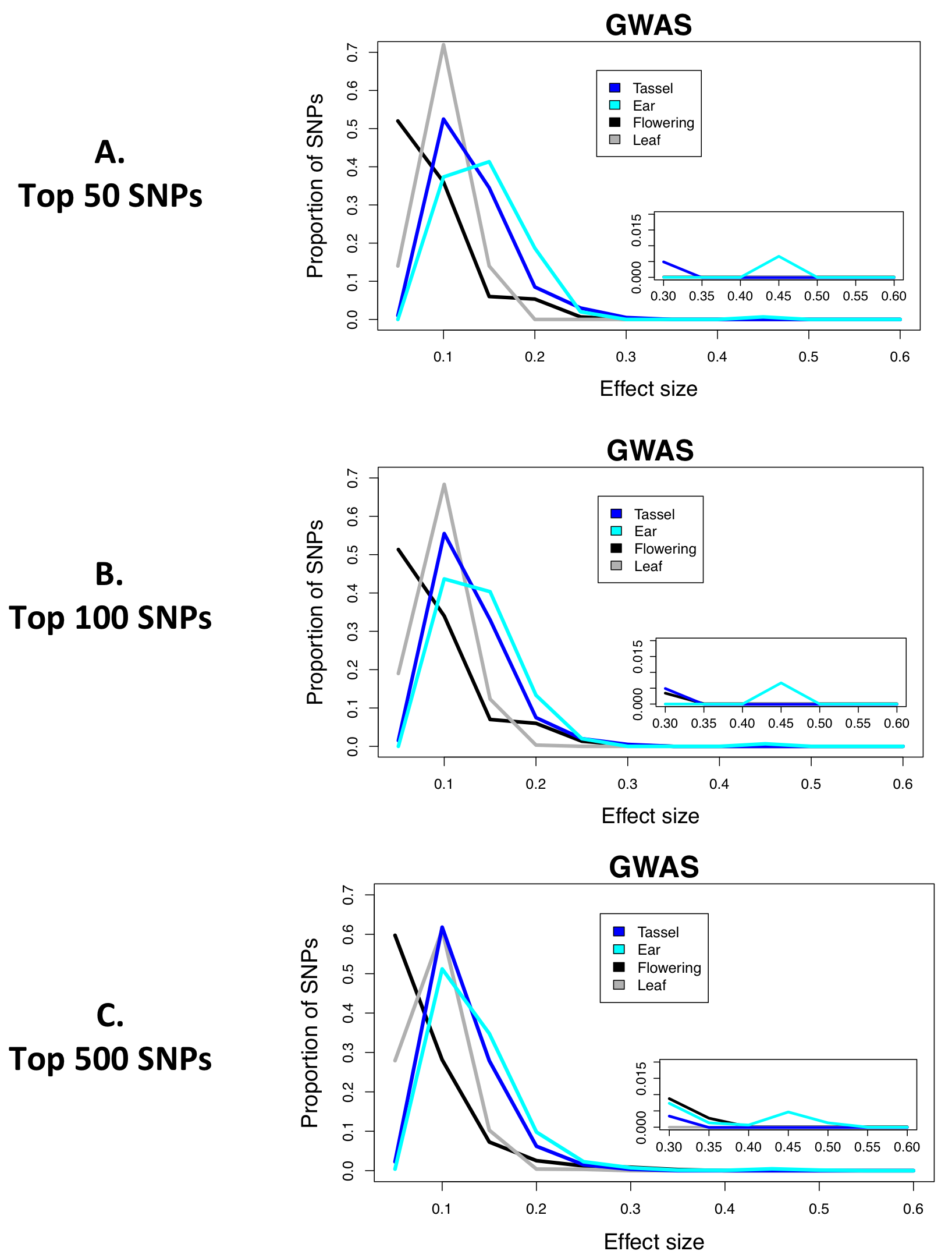

Supplement: Figure S3 — Frequency distributions for scaled GWAS effects produced using the top 50 (A), 100 (B), and 500 (C) SNPs for each trait. Results are similar to those presented at the bottom of Figure 2. (TIF) [file pgen.1002383.s003.tif]

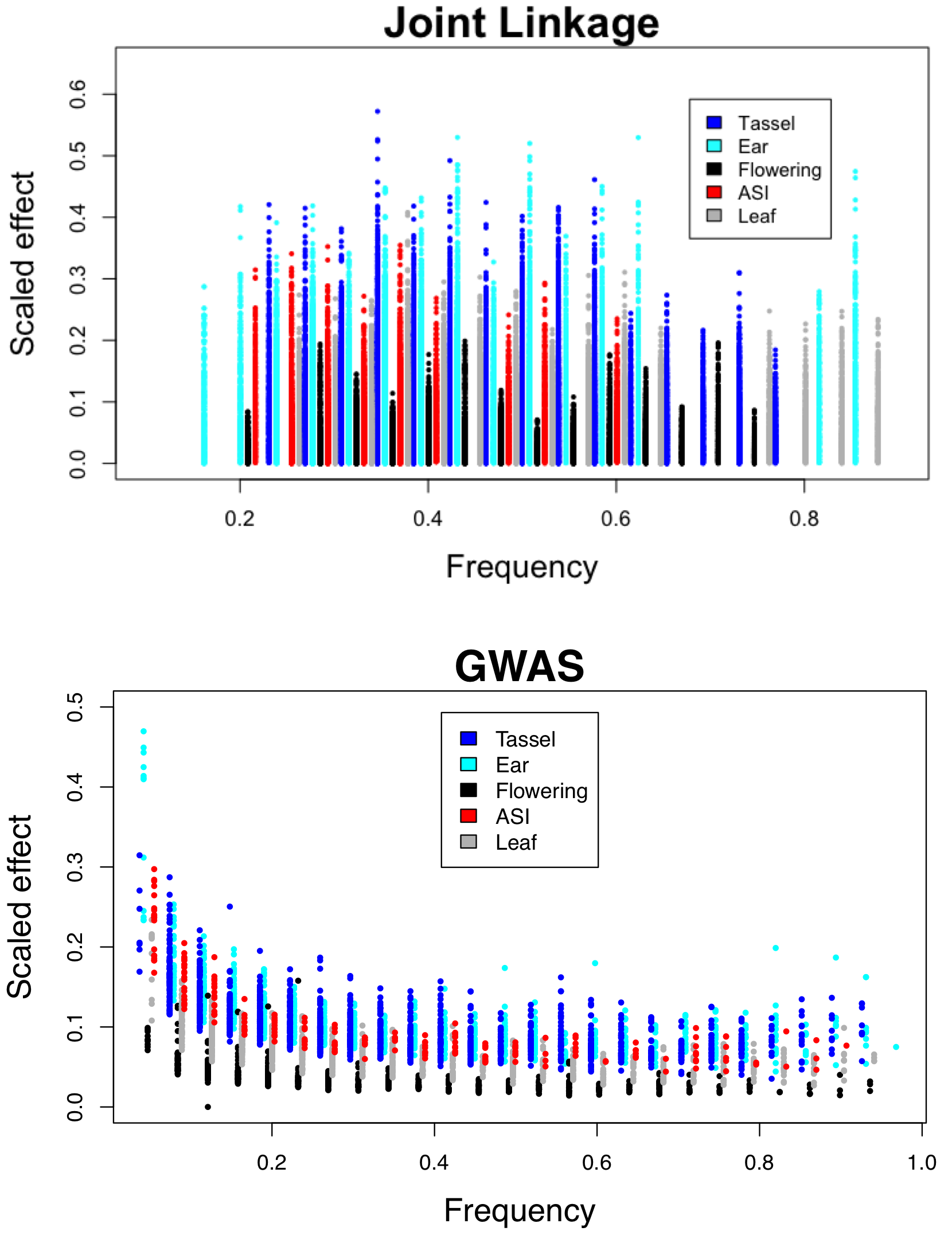

Supplement: Figure S4 — Relationship between QTL frequency and scaled QTL effects in the joint linkage (A) and GWAS (B) analyses, as in Figure 3 but with ASI effects colored in red. (TIF) [file pgen.1002383.s004.tif]

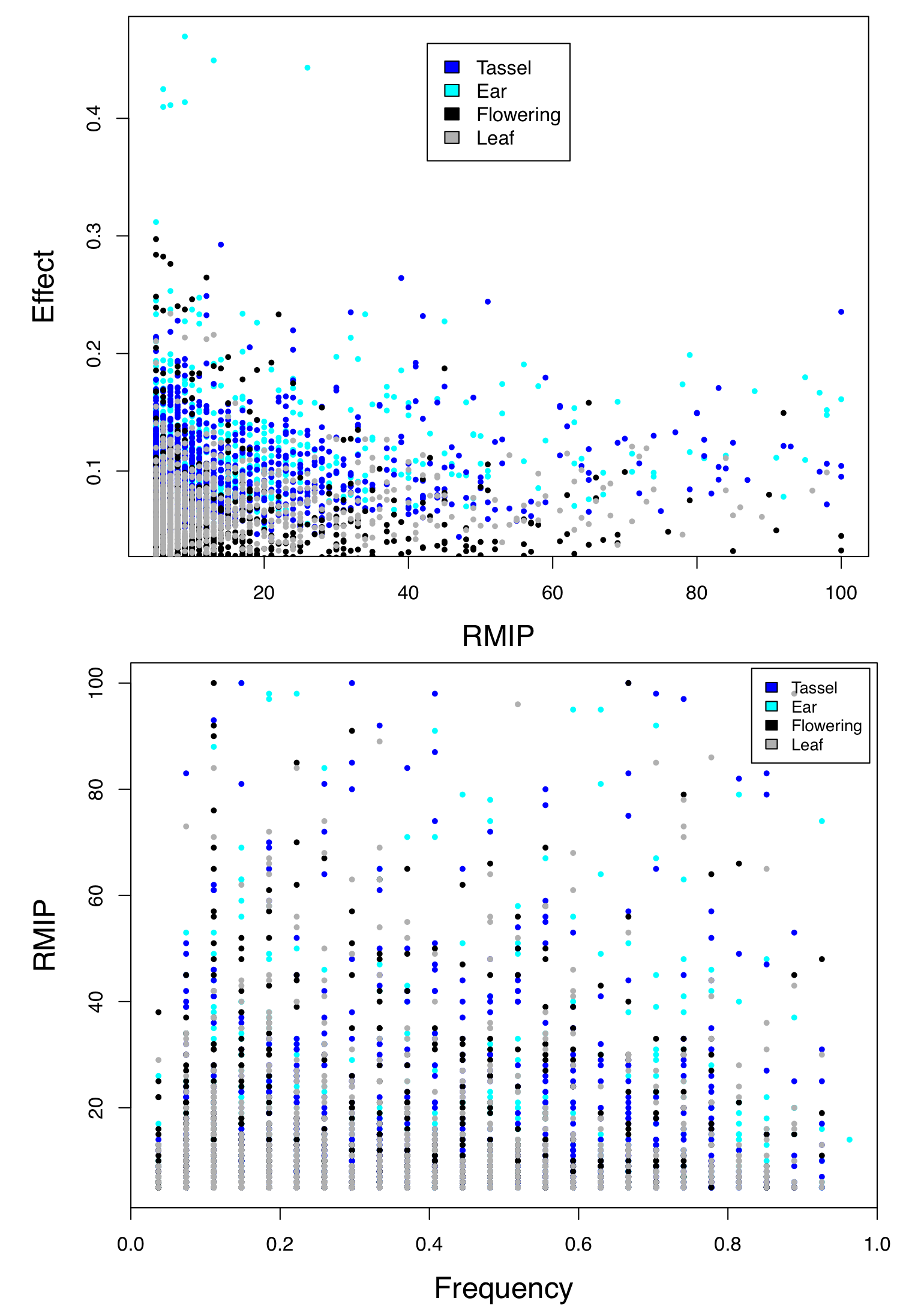

Supplement: Figure S5 — Relationship between RMIP and scaled effects (top), and between SNP frequency and RMIP (bottom). (TIF) [file pgen.1002383.s005.tif]

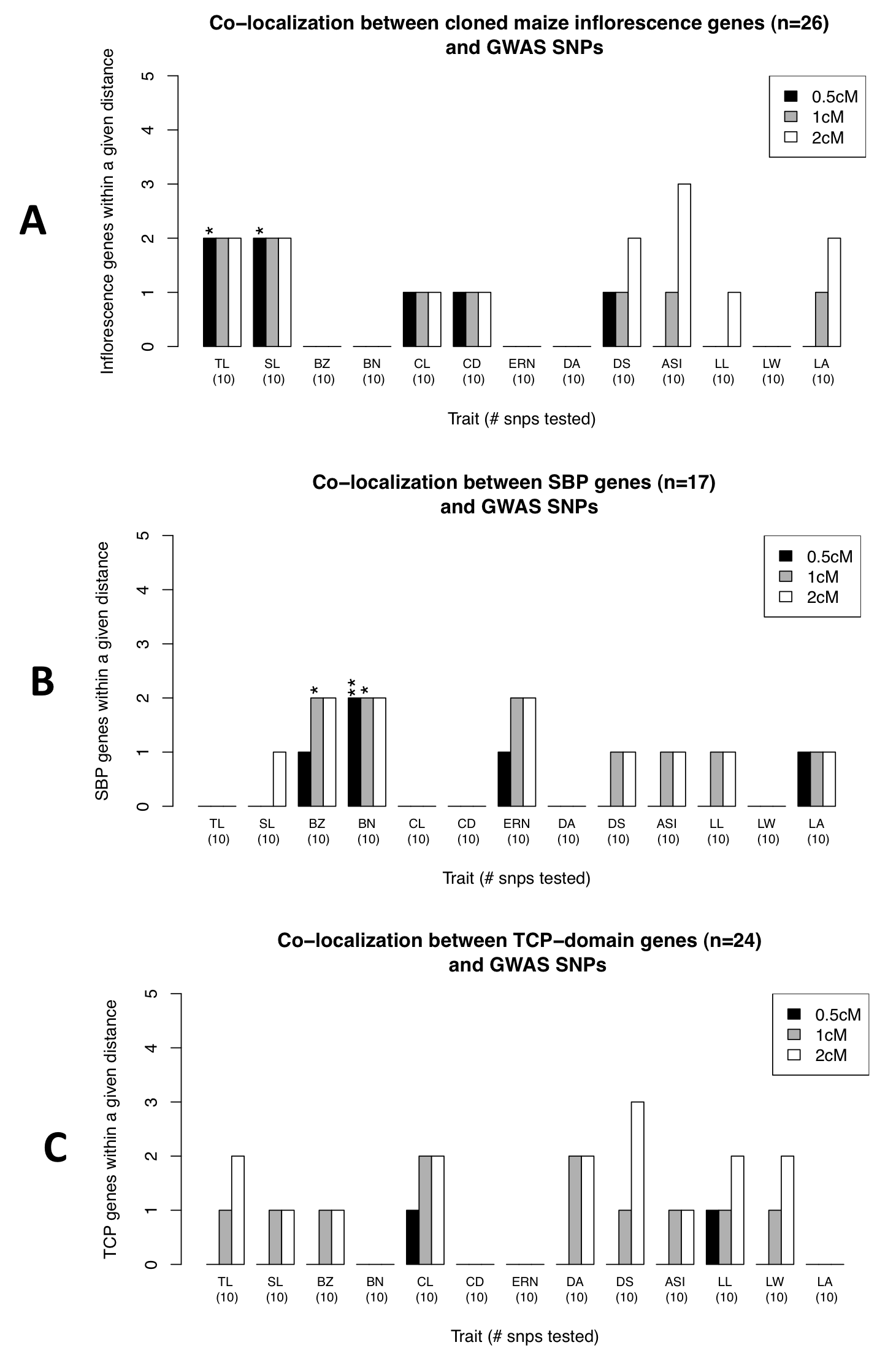

Supplement: Figure S6 — Co-localization of GWAS SNPs and candidate genes. A: cloned inflorescence mutants (n = 26). B: SBP-domain genes (n = 17). C: TCP-domain genes (n = 24). The 10 GWAS SNPs with the highest RMIP values were tested for each of 13 maize morphological traits. The number of GWAS SNPs falling within 0.5, 1, and 2 cM of a candidate gene is indicated with black, grey, and white bars respectively. Significance at levels of p<0.05 (*) and 0.01(**) was obtained by selecting an equal number of random maize genes to the number of candidates, calculating their genetic distances to the top 10 GWAS SNPs, and repeating this procedure 1000 times. Significance levels differ greatly between traits due to differences in the genetic context of their GWAS SNPs. SNPs in regions of low recombination may fall within 1 cM of many more random genes than SNPs in regions of high recombination. (TIF) [file pgen.1002383.s006.tif]
